# First field-based observations of *δ* ^2^H and *δ* ^18^O values of event-based precipitation, rivers and other water bodies in the Dzungarian Gobi, SW Mongolia

**DOI:** 10.1080/10256016.2016.1231184

**Published:** 2016-10-12

**Authors:** Martina Burnik Šturm, Oyunsaikhan Ganbaatar, Christian C. Voigt, Petra Kaczensky

**Affiliations:** ^a^ Research Institute of Wildlife Ecology, University of Veterinary Medicine Vienna, Vienna, Austria; ^b^ Great Gobi B Strictly Protected Area Administration, Takhin Tal, Gobi Altai Province, Mongolia; ^c^ School of Biology and Biotechnology, National University of Mongolia, Ulan Bator, Mongolia; ^d^ Department of Evolutionary Ecology, Leibniz Institute for Zoo and Wildlife Research, Berlin, Germany

**Keywords:** Central Asia, deuterium excess, extreme environment, Gobi desert, hydrogen-2, isotope hydrology, oxygen-18, water cycle

## Abstract

For certain remote areas like Mongolia, field-based precipitation, surface and ground water isotopic data are scarce. So far no such data exist for the Mongolian Gobi desert, which hinders the understanding of isotopic fractionation processes in this extreme, arid region. We collected 26 event-based precipitation samples, 39 Bij river samples, and 75 samples from other water bodies in the Dzungarian Gobi in SW Mongolia over a period of 16 months for hydrogen and oxygen stable isotope analysis. *δ* ^2^H and *δ* ^18^O values in precipitation show high seasonal variation and cover an extreme range: 175 ‰ for *δ* ^2^H and 24 ‰ for *δ* ^18^O values. The calculated local meteoric water line (LMWL) shows the isotopic characteristics of precipitation in an arid region. Individual water samples fall into one of three groups: within, above or below the 95 % confidence interval of LMWL. Data presented provide a basis for future studies in this region.

## 1. Introduction

Hydrogen (*δ* ^2^H) and oxygen (*δ* ^18^O) isotope values of water are widely used for tracing the global hydrogeological cycle, and a derived isotopic parameter, namely the *d*-excess (*d* = *δ* ^2^H − 8 *δ* ^18^O; [1]), is used to study moisture recycling processes [2,3]. Local meteoric water lines (LMWLs), calculated from *δ* ^2^H and *δ* ^18^O values of local precipitation, are an important part of groundwater investigations that compare isotopic ratios in groundwater or surface water with precipitation at specific locations [4], and to determine the evaporative enrichment of local water bodies. Moreover, over the last decade, global hydrogen and oxygen isotopic patterns of precipitation have increasingly been used in animal migration [5–9], forensic [10–12], and food authentication and traceability studies [13–16]. However, records of the stable isotope composition of precipitation spanning one or more years are available for only a few hundred locations worldwide [17], and data for Mongolia are especially scarce. Yumanaka et al. [18] report data for eastern Mongolia (October 2002 to September 2003), and Schotterer et al. [19] report ice cap core data from Tsast Ula in north-western Mongolia (covering 20 years), but to our knowledge, no such data exist for the southwestern arid parts, i.e. the Dzungarian Gobi desert. The lack of availability of field-based isotopic data significantly hinders the understanding of isotopic fractionation processes in this extreme, arid region. The nearest station contributing data for long-term International Atomic Energy Agency (IAEA) Global Network of Isotopes in Precipitation (GNIP) is the Fukang station (87°56′0″E, 44°16′60″N, 460 m above sea level (a.s.l.)) in NW China, located over 450 km south-west from our study area, i.e. the Gobi B Strictly Protected Area (SPA) in the Dzungarian Gobi in Mongolia, from which it is separated by a mountain range at the international border between China and Mongolia.

We aimed at closing this gap by: (1) collecting samples of event-based precipitation, weekly river samples, spring and other surface water samples in the Great Gobi B SPA and adjacent parts in the Dzungarian Gobi in SW Mongolia over a period of 16 months (June 2012 to September 2013) for stable hydrogen and oxygen isotope analysis, (2) establishing the LMWL, and (3) obtaining baseline information on *δ* ^18^O and *δ* ^2^H values of precipitation, river and other water bodies in this area.

## 2. Materials and methods

### 2.1. Study area

**Figure 1.**
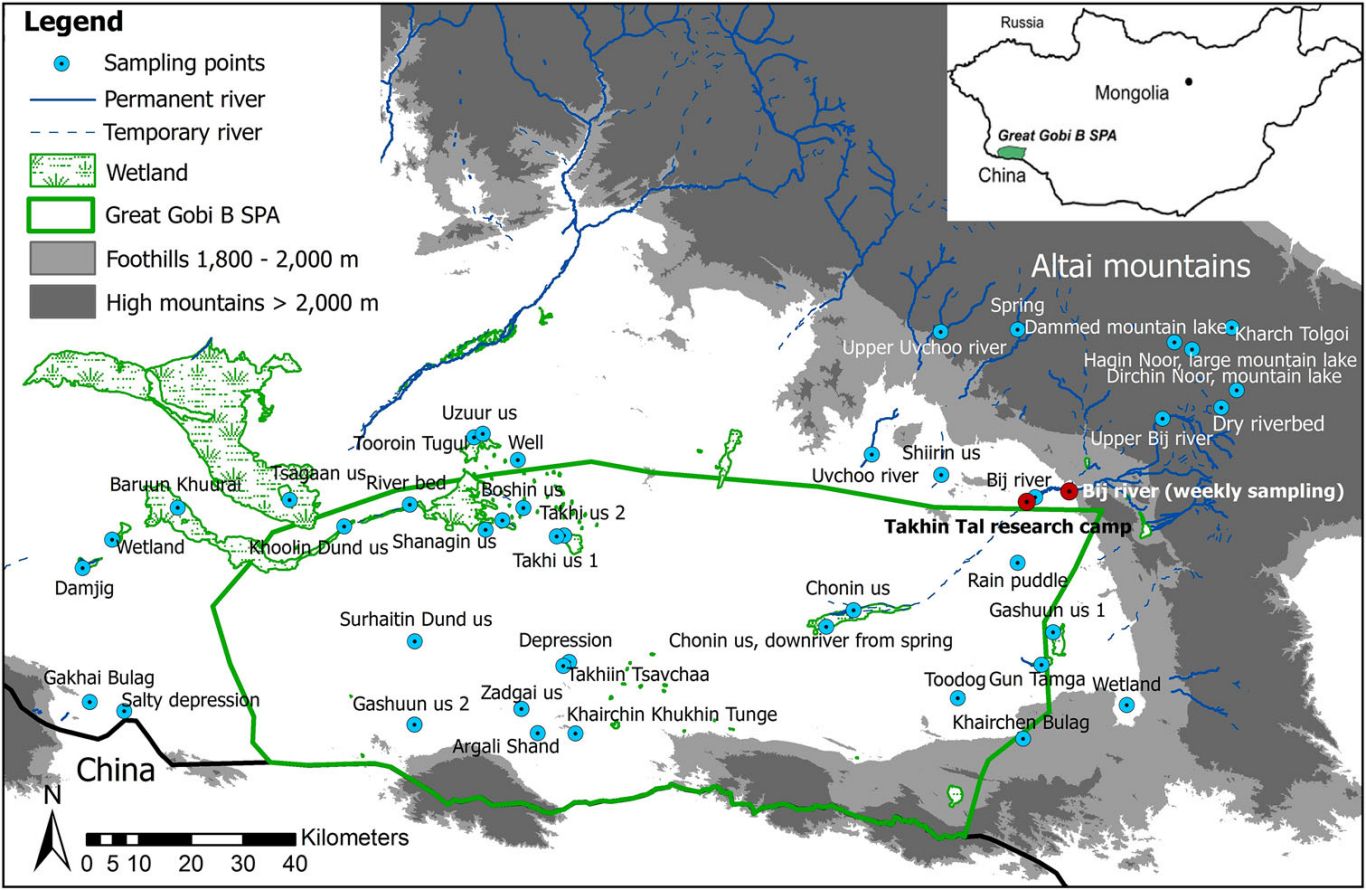
Sampling locations in and around the great Gobi B SPA in the Dzungarian Gobi, southwestern Mongolia. Precipitation samples were collected at the Takhin Tal research camp.

The Great Gobi B SPA stretches over 9000 km^2^ and covers a large part of the Dzungarian Gobi in southwestern Mongolia (Figure 1) [20,21]. In the east, the landscape of the Great Gobi B SPA is dominated by plains, in the west by rolling hills and in the south by mountains, which form the international border with China. To the north, the Altai Mountains form the northern border of the Dzungarian basin. Elevations within the SPA range from 1000 to 2840 m a.s.l.. The climate of the study area is strongly continental with monthly temperatures averaging +3.4 °C in spring (March–May), +17.9 °C in summer (June–August), +1.6 °C in fall (September–November) and −17.9 °C in winter (December–February) and with extremes ranging from +35 to −43 °C (data 2003–2012, Takhin Tal research camp at 45°32′12″N/93°39′05″E and 1760 m a.s.l., HOBO temperature logger, Hoskin Scientific Limited, Vancouver, Canada).

**Figure 2.**
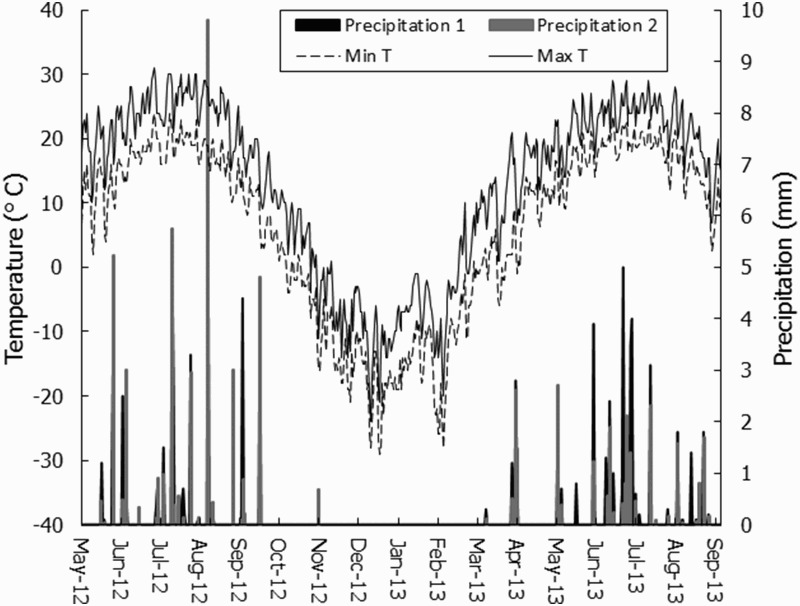
Weather data for the study period obtained from the Global Livestock Early Warning System (GLEWS) for two individual sites (Precipitation 1 and 2, about 35 km apart) in relative proximity to the research camp (∼100 and 70 km distance, respectively), demonstrating high spatial variability in the precipitation (temperature difference between the two sites is minimal).

Average rainfall is 100 mm with a distinct peak in summer (>80 %; June–August; WorldClim 1 km resolution [22]). Average snow cover lasts for around 100 days. Rain and snowfall are highly variable in space and time (Figure 2). Due to western disturbances that pass the Turanic highlands, snowfall seems to be more intense compared to the rest of the southern Mongolian Gobi, which is under the influence of the East-Asian Monsoon [23,24].

Open water (i.e. rivers and springs) is unevenly distributed with little water in the central and western part of the park. In locations where several springs occur together, they are surrounded by intermittent wetlands and form oases. Springs are largely permanent, although their flow varies throughout the year and they freeze up in winter.

Most hills and mountains are built of metamorphic deep-sea sediments; parts of the highest mountains are built of granite rocks. Sand covers large parts of the northern hilly regions and the soils are shallow and often poorly developed [24].

### 2.2. Sample collection

Between June 2012 and September 2013, we collected 26 event-based precipitation samples at the Takhin Tal research camp (45°32′12″N, 93°39′05″E; 1760 m a.s.l.) located at the edge of the Gobi B SPA, 39 Bij river samples, taken weekly at the same site (45°33′22″N, 93°45′23″E; 1765 m a.s.l.), and 75 samples of various additional water bodies inside and around of the Great Gobi B SPA (Figure 1, Table 1, Table SM 1 and 2). Precipitation samples were collected from a rain collector (HDPE sampling bottle with a plastic funnel) as soon as possible after a precipitation event to avoid evaporation, transferred into 50 ml bottles with a tightly fitting cap, and kept in a dark place until transport and analysis. Snow samples were melted in a covered sampling container at room temperature before transferring to 50 ml bottles. Identical bottles and storing procedure were used for all other water samples.

**Table 1.** *δ* ^2^H and *δ* ^18^O values of various water bodies, sampled in great Gobi B SPA and adjacent areas in the period between June 2012 and August 2013.

Sample	Date	*x*_co_	*y*_co_	Elevation (m a.s.l.)		*δ*^18^O (‰)	*δ*^2^H (‰)	*d*-excess
Takhi us 1, water point, wetland	17 June 2012	92.51429	45.48184	1227		−14.2	−131	−17
	24 July 2012	92.51429	45.48184	1227		−12.2	−123	−26
	13 August 2013	92.51429	45.48184	1227		−12.2	−123	−25
Takhi us 2, wetland, shallow pools	20 June 2012	92.49603	45.48013	1226		−14.6	−134	−17
Argali Shand, wetland	17 June 2012	92.45261	45.13961	1703		−5.9	−88	−40
Tooroin Tugul, wetland	20 June 2012	92.29084	45.65011	1147		−15.4	−134	−11
Uzuur us, wetland	20 June 2012	92.31266	45.65646	1151		−13.0	−127	−24
Tsagaan us, main water point of large wetland	19 June 2012	91.83917	45.53840	1094		−11.7	−117	−24
Damjig, large wetland	19 June 2012	91.33363	45.41427	1186		−17.0	−140	−4
Large wetland	19 June 2012	91.40449	45.46449	1133		−16.8	−146	−12
Gashuun us 1	22 June 2012	93.71385	45.31341	1659		−18.2	−157	−11
	14 April 2013	93.71385	45.31341	1659		−18.0	−156	−12
	15 June 2013	93.71385	45.31341	1659		−18.3	−157	−10
	12 August 2013	93.71385	45.31341	1659		−18.3	−156	−9
Gashuun us 2, wetland with fresh pools	18 June 2012	92.15154	45.15259	1666		−15.9	−140	−13
	16 April 2013	92.15154	45.15259	1666		−17.2	−148	−10
	18 June 2013	92.15154	45.15259	1666		−16.1	−142	−14
	13 August 2013	92.15154	45.15259	1666		−12.7	−126	−25
Gashuun us 3, wetland with small pools	20 June 2012	92.41369	45.52901	1184		−11.9	−124	−28
	24 July 2012	92.41369	45.52901	1184		−16.8	−143	−9
Wetland, W83	02 July 2012	93.89219	45.18604	1768		−10.1	−121	−40
Bij river	12 June 2012	93.67251	45.54700	1702		−16.1	−138	−10
Upper Bij river, in mountains before the dam	27 June 2012	93.98796	45.68054	2257		−16.4	−137	−6
Ovchuu river	20 June 2012	93.27103	45.62225	1635		−18.0	−153	−9
	24 July 2012	93.27103	45.62225	1635		−17.5	−149	−9
	17 April 2013	93.27103	45.62225	1635		−17.1	−148	−11
	15 August 2013	93.27103	45.62225	1635		−18.1	−154	−9
Upper Ovchuu river	26 June 2012	93.44210	45.83409	2002		−18.6	−155	−7
Dammed mountain lake	27 June 2012	94.02000	45.81199	2801		−8.3	−101	−35
Khagin nuur, large mountain lake	27 June 2012	94.06293	45.79938	2605		1.0^a^	−37	−45
Dirchin nuur, mountain lake	27 June 2012	94.13357	45.69802	2714		−5.4	−77	−35
Depression with 3 holes, digging by khulan	17 June 2012	92.52825	45.26294	1617		−11.3	−120	−29
Well, spring herder camp	20 June 2012	92.39906	45.61166	1162		−17.2	−147	−9
Khairchin Khukhin Tunge, old broken well	17 June 2012	92.54503	45.13986	1719		−6.9	−105	−50
Takhiin Tsavchaal, broken well	17 June 2012	92.51457	45.25644	1643		−13.3	−125	−18
Baruun Khuurai, dried up, rain water standing	19 June 2012	91.56396	45.52187	1076		7.0^a^	−15	−71
Khoolin Dund us, river bed with pools	19 June 2012	91.97418	45.49406	1103		−13.1	−123	−19
River bed with pools	20 June 2012	92.13435	45.53263	1127		−7.0	−98	−42
Gakhai bulag, small spring in mountains	18 June 2012	91.35791	45.18326	1457		−9.0	−101	−29
Zadgai us, water point in the herder camp	17 June 2012	92.41294	45.18167	1704		−18.3	−157	−11
	24 July 2012	92.41294	45.18167	1704		−18.1	−157	−12
	17 April 2013	92.41294	45.18167	1704		−16.4	−144	−13
	18 June 2013	92.41294	45.18167	1704		−18.1	−156	−11
	13 August 2013	92.41294	45.18167	1704		−18.0	−156	−12
Surhaitin Dund us, water hole	18 June 2012	92.15012	45.29674	1436		−12.5	−125	−25
Bosgo us, spring with river, several pools	20 June 2012	92.36254	45.50712	1170		−8.1	−105	−40
	24 July 2012	92.36254	45.50712	1170		−5.0	−93	−53
	16 April 2013	92.36254	45.50712	1170		−16.0	−141	−13
	18 June 2013	92.36254	45.50712	1170		−15.7	−142	−16
	13 August 2013	92.36254	45.50712	1170		−12.5	−128	−28
Shanagin us	20 June 2012	92.32127	45.49047	1163		−4.9	−88	−49
Shiiriin us	16 June 2012	93.44181	45.58680	1657		−17.7	−151	−9
	24 July 2012	93.44181	45.58680	1657		−18.5	−155	−7
	15 April 2013	93.44181	45.58680	1657		−15.5	−134	−10
	18 June 2013	93.44181	45.58680	1657		−17.7	−152	−10
	15 August 2013	93.44181	45.58680	1657		−18.4	−154	−7
Guntamga	12 June 2012	93.68456	45.25739	1636		−19.4	−164	−9
	14 April 2013	93.68456	45.25739	1636		−19.8	−168	−9
	16 August 2013	93.68456	45.25739	1636		−18.5	−161	−13
Toodog	11 June 2012	93.47908	45.20050	1657		−15.7	−144	−19
Rain puddle	12 June 2012	93.62820	45.43390	1614		−10.5	−96	−12
Khonin us, downriver from spring	22 June 2012	93.15847	45.32514	1396		−12.3	−128	−29
Khonin us	22 June 2012	93.22495	45.35328	1413		−17.4	−146	−7
	27 July 2012	93.22495	45.35328	1413		−16.7	−151	−17
	15 April 2013	93.22495	45.35328	1413		−17.2	−146	−9
	16 June 2013	93.22495	45.35328	1413		−16.8	−151	−16
	13 August 2013	93.22495	45.35328	1413		−16.8	−151	−16
Little spring/river	26 June 2012	93.63226	45.83689	2208		−20.0	−165	−5
Kharch Tolgoi, spring	27 June 2012	94.16200	45.83591	2488		−15.9	−145	−17
Dry riverbed with pools	27 June 2012	94.17175	45.72777	2647		−15.1	−134	−13
Water in salty depression	18 July 2012	91.44225	45.16854	1201		−5.3	−82	−40
Khairkhan bulag, spring	02 July 2012	93.63847	45.12986	1816		−17.6	−154	−14
	14 April 2013	93.63847	45.12986	1816		−17.2	−151	−13
	15 June 2013	93.63847	45.12986	1816		−17.7	−155	−14
	12 August 2013	93.63847	45.12986	1816		−17.5	−154	−15
								

^a^ *δ* ^18^O values of a sample exceed the range of the reference materials used for the analysis.

### 2.3. Weather data

Due to technical problems with the weather station at the research camp, we were not able to collect own weather data during the course of the study. Instead, we obtained modelled daily precipitation and temperature data for two locations in the relative proximity (∼100 and 70 km, respectively) to the research camp (i.e. Precipitation 1: HO-0040, 45°18′27″N/92°24′05″E, and Precipitation 2: HO-0041, 45°29′10″E/92°46′11″E; Figure 2) from the GLEWS [25].

### 2.4. Back-trajectory analysis

To determine the source of air masses bringing precipitation, we ran air mass back-trajectory analysis for each of the collected 26 samples, using the HYSPLIT model (HYbrid Single-Particle Lagrangian Integrated Trajectory model, online version: http://ready.arl.noaa.gov/HYSPLIT.php) [26], developed by the National Oceanographic and Atmospheric Administration (NOAA). The HYSPLIT model uses a three-dimensional Lagrangian air mass velocity algorithm to determine the position of the air mass. It calculates air mass position through time using pressure, temperature, wind speed, vertical motion and solar radiation inputs from the NOAA meteorological database (e.g. GDAS, global data assimilation system; archived data: 2006–present). Back-trajectories were modelled for 168 hours (7 days) before the time each sample was collected, with a start time of 21 UTC. Starting location was the sampling site. Trajectories originated at 1500, 2000 and 3000 m above ground level (a.g.l.).

### 2.5. Stable isotope analysis

The stable isotope abundances of water samples were analysed at the isotope facility of the IZW Berlin, using a Piccaro L1102-i water analyser (Piccaro, Santa Clara, CA, USA). Water samples were introduced into the vaporization chamber with the injection port using an attached PAL autosampler (CTC Analytics AG, Zwingen, Switzerland). Approximately 1 μl of water sample was injected into a heated vaporizer (140 °C) and then transferred to the cavity of the spectroscopic analyser where isotopologue concentrations were determined by cavity ring-down spectroscopy. Three international reference materials (SLAP, GISP and VSMOW) and an additional in-house reference material (calibrated against VSMOW-SLAP scale) were included in each batch to correct the raw values via three-point regression line (SLAP, GISP and VSMOW). The results are expressed in delta per mil notation (*δ*, ‰) relative to the international standard VSMOW. Precision of the measurements was better than 1.4 ‰ for *δ* ^2^H and 0.3 ‰ for *δ* ^18^O. The *d*-excess (*d* = *δ* ^2^H − 8*δ* ^18^O, [1]) was calculated for all samples.

We used R (version 3.1.1, R development Core Team, 2014 [27]) for calculation of the linear regression lines and respective 95 % confidence intervals (CIs), and excel spreadsheets for calculations of *d*-excess.

## 3. Results and discussion

### 3.1. Precipitation isotope pattern and the LMWL

**Figure 3.**
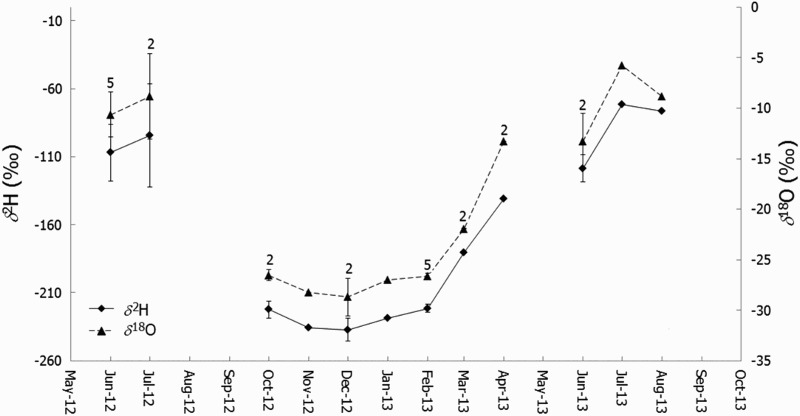
Isotopic distribution of *δ* ^2^H and *δ* ^18^O values of precipitation, collected in the great Gobi B SPA in SW Mongolia (45°32′12″N, 93°39′05″E; 1760 m a.s.l.). Numbers above error bars represent the number of analysed samples for the months with several precipitation events (see Table SM 1 for *δ* ^2^H and *δ* ^18^O values of individual events).

During the study period, 26 precipitation events were recorded and sampled at the Takhin Tal research camp (Figure 3, Table SM 1; as previously reported in [28]), among these, 13 were snow events, that occurred between October 2012 and March 2013.

**Figure 4.**
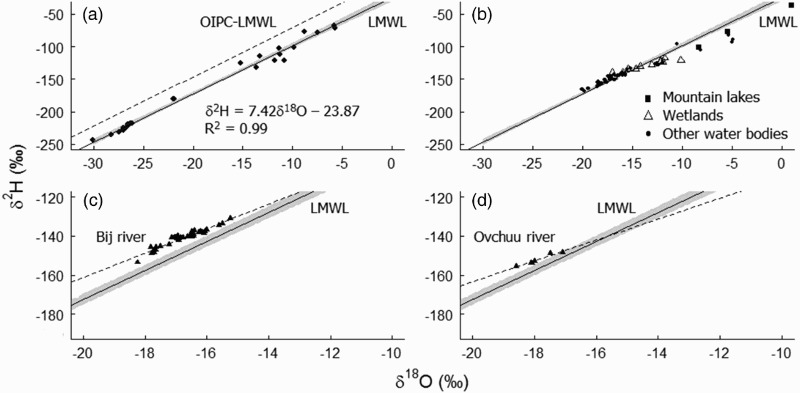
Field based *δ* ^2^H and *δ* ^18^O values in (a) precipitation with the corresponding LMWL (solid line) with 95 % CI (grey envelope; *n* = 26) and LMWL, calculated using OIPC calculator (dotted line), (b) various water bodies (*n* = 75), (c) Bij river (*n* = 39) and (d) Ovchuu river (*n* = 5), plotted against LMWL. Dotted lines in figures c and d represent respective river linear regression lines. Note the different *δ* ^2^H and *δ* ^18^O scales in figures a, b and c, d, respectively.

Precipitation data from the study area show an extreme seasonal variability in stable isotope values. Water of summer precipitation (precipitation dominant season) is more enriched in heavier isotopes and more variable as compared to that of winter precipitation (Figures 3 and 4), which is in line with findings of Wang et al. [29], who studied distribution and seasonal variability in arid conditions in the Tianshan Mountains in China over a similar sampling period (August 2012–September 2013). Overall, *δ* ^2^H values range from −243 to −68 ‰ (Δ = 175 ‰) and *δ* ^18^O values from −30 to −6 ‰ (Δ = 24 ‰). Comparable mean amount-weighted precipitation isotope values, namely *δ* ^2^H = −199 ‰ and *δ* ^18^O = −71 ‰ in winter (*n* = 2), and *δ* ^2^H = −28 ‰ and *δ* ^18^O = −10 ‰ in summer (*n* = 21), have been reported for the sampling station Yiwu (M6), China, located 266 km south from our study site, with similar elevation (1728 m a.s.l.) and average precipitation (104.4 mm) to our study area [29]. A much wider range in *δ* ^2^H (−179.9 to + 51.9 ‰) and *δ* ^18^O (−25.7 to + 8.5 ‰) values has been reported by Sun et al. [30] for the Tarim River Basin, China, which is ascribed to high summer temperatures and very strong evaporation in this extremely dry desert climate with one of the highest aridity indexes (ratio of annual potential evaporation to precipitation) in the world [30].

We calculated the LMWL, based on event data, to be *δ* ^2^H = (7.42 ± 0.16)*δ* ^18^O – (23.87 ± 3.27), and clearly identified a significant linear correlation (*R* ^2^ = 0.99, *n* = 26) between *δ* ^2^H and *δ* ^18^O values (Figure 4(a)). The slope and intercept of the linear regression line were lower compared to the global meteoric water line [31], the regional meteoric water line for Southeast Asia [32], as well as the LMWL determined for the Tianshan Mountains in Central Asia (event-based data) [29], and show the isotopic characteristics of precipitation in an arid region [4,29,33,34]. The regional meteoric water line of eastern Mongolia [18] has a very similar slope (7.40), but a significantly higher intercept (1.1) compared to our LMWL*.* In our samples, *d*-excess values were much lower than the global average of ∼10 ‰, and ranged between −3 and −35 ‰ (Table SM 1). We found less depleted values for snow (mean *d*-excess = −9 ± 3 ‰) than for rain samples (mean *d*-excess = −18 ± 10 ‰). According to literature [2,29,30,33,35], isotope values below the GMWL signify the effect of subcloud evaporation during summer season, i.e. high *δ* ^18^O and low *d*-excess values indicate that the isotopic enrichment due to subcloud evaporation over-compensates the isotopic depletion by moisture recycling. *d*-excess values below 0 ‰ have also been reported for Venezuela, Mali and Chad, for stations with high temperature and/or low vapour pressure, where kinetic isotope fractionation processes, connected with the partial evaporation of the falling raindrops [35 in 36], affected the *d*-excess of the precipitation [37].

Compared to our field-based LMWL, the LMWL calculated using the Online Isotopes in Precipitation Calculator (OIPC; *δ* ^2^H = (7.60 ± 0.14)*δ* ^18^O + (5.18 ± 2.26); Figure 4(a), Table SM 1) [17,38,39], which uses modelled *δ* ^2^H and *δ* ^18^O values for the studied area, lies outside and to the left of 95 % CI of our field-based LMWL and thus somewhat underestimates the evaporative processes for this region. The OIPC-calculated *d*-excess values were also considerably higher compared to *d*-excess, calculated from the field-based isotope data. The lack of long-term GNIP data across large portions of the broader region, as well as extreme seasonality and high variability in precipitation in time and space, characteristic for the study area, can probably explain the large differences between field based and modelled (OIPC) data and thus between the field based and modelled LMWL.

**Figure 5.**
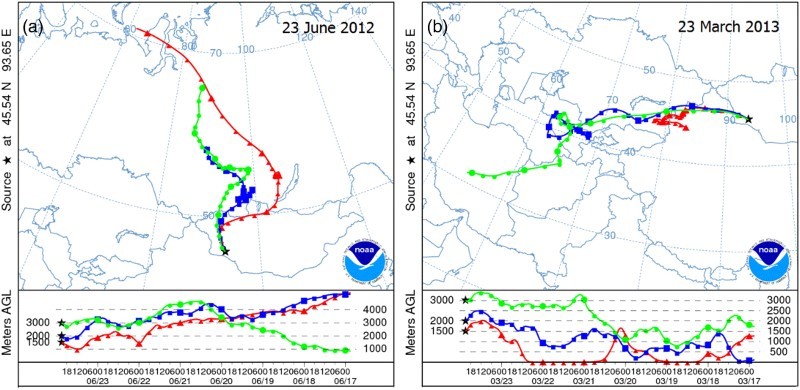
7-Day back-trajectories calculated by HYSPLIT model at 1500 m (red lines), 2000 m (blue lines) and 3000 m (green lines) a.g.l., representing (a) northern polar winds and (b) westerly winds, ending at the Takhin Tal research camp, Gobi B SPA, Mongolia.

Mongolia is a landlocked country located in the centre of the Asian continent. With air masses traveling from the sea to the interior of the continent, they gradually bring increasingly isotopically depleted precipitation as a consequence of the so called continental effect [32]. *d*-excess values are often interpreted as indicators of the origin of air masses, but due to evaporative isotopic effects, such interpretations are problematic in arid regions [40]. The 7-day back-trajectories, simulated by the HYSPLIT model, indicate that air masses were mainly carried to the study site from north/north-west by polar winds over Russia (e.g. Figure 5(a)) and by westerlies over Central Asia (e.g. Figure 5(b)). The variability in the origin of air masses (i.e. polar/westerly) was most pronounced during summer, which coincides with the highest variability in isotope values among individual summer rain events (as indicated by high deviations from the LMWL 95 % CI in summer precipitation; Figure 4(a)). However, high variability in isotope values could not be explained by different origin of air masses alone. While in some cases the difference in isotope values between individual precipitation events is supported by different air mass trajectories (e.g. 19 June 2013 and 30 July 2013, with northern and westerly winds, respectively, Δ*δ* ^18^O = 5.5 ‰; not shown), we found an almost 8 ‰ difference in *δ* ^18^O values between consecutive rain events with similar trajectories (22 and 23 June 2012, northern winds; not shown), indicating that additional fractionating processes had taken place.

Temperature has been recognized as an important factor affecting isotope values of precipitation, with a positive relationship between *δ* ^18^O and *δ* ^2^H and temperature (i.e. temperature effect) [1]. As the study area in the Dzungarian Gobi is characterized by cold winters, hot summers (Figure 2) and extreme daily temperature swings that can range from frost at dawn to over +30 °C at noon, it is likely that, at least in part, high inter- and intra-seasonal variability of isotope values can be explained by these high temperature fluctuations. This is in agreement with Araguas-Araguas et al. [32], who studied isotope composition of precipitation over southeast Asia and found that *δ* ^18^O and *δ* ^2^H of rainfall at latitudes above approximately 35°N are mainly controlled by temperature and less by the amount effect (i.e. negative relationship between *δ* ^18^O and *δ* ^2^H and amount of precipitation). Li et al. [41] also found that in China, at latitudes above 32°N, temperature has the main effect on isotope values, but only during winter, whereas in summer, the amount effect plays a more important role. Wang et al. [29] found that in addition to temperature, the seasonal distribution of precipitation (in our study area, on average 80 % of all precipitation falls during summer) also greatly influences the isotopic composition of precipitation in arid regions.

Due to the lack of data on precipitation amount, we were not able to evaluate the importance of the amount effect on the isotope values of precipitation for our study area. A detailed study of various meteorological parameters (e.g. relative humidity, actual air temperature, solar radiation, pressure) would additionally be needed to better understand the processes affecting the isotopic values of precipitation in the study area.

### 3.2. Rivers and other water bodies

#### 3.2.1. Bij and Ovchuu river

**Figure 6.**
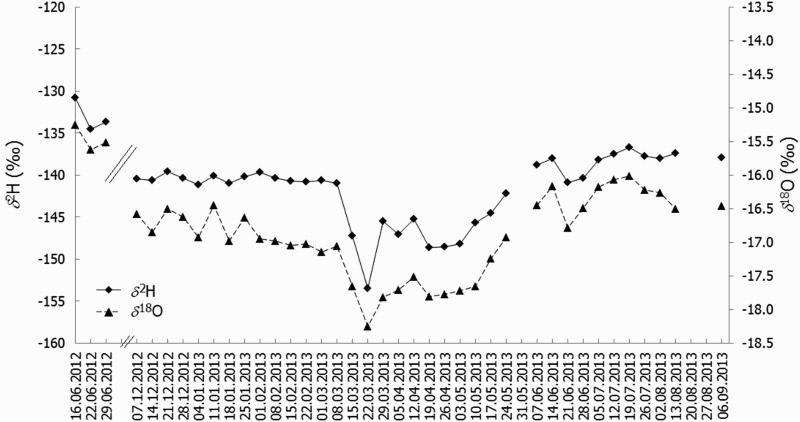
Weekly *δ* ^2^H and *δ* ^18^O values of Bij river, collected between June 2012 and September 2013 in the great Gobi B SPA in SW Mongolia (45°33′12″N, 93°45′05″E; 1765 m a.s.l.; Table SM 2).

Flow of the Bij river is highly variable, depending mostly on water originating from rainfall and snowmelt events in the upstream catchment high in the mountains where the river emerges. This is reflected in Bij river *δ* ^2^H and *δ* ^18^O values (*δ* ^2^H = (6.26 ± 0.31)*δ* ^18^O – (35.92 ± 5.15), *R* ^2^ = 0.92, *n* = 39; Table SM 2) which fall left to and outside of the 95 % CI of the LMWL (Figure 4(c)). Despite the exceptionally large isotopic variations observed in precipitation in the valley (Figure 3), valley precipitations do not seem to have a large effect on the river values (Figure 6). The temporal variation of *δ* ^2^H and *δ* ^18^O values of Bij river water was small over the entire course of our study (i.e. −22.7 ‰ for *δ* ^2^H and −3 ‰ for *δ* ^18^O values; Figure 6). The *d*-excess values ranged between −2.9 and −9.6 ‰ (mean *d*-excess = −6 ± 2 ‰, Table SM 2).

Slightly more negative *δ* ^2^H and *δ* ^18^O values of Bij river water between March and May correspond to the inflow of snowmelt; whereas the slightly more positive values observed between June and September suggest evaporative enrichment. Similar temporal variation is reported for the Kaidu river in NW China [42] and the Gunt river in the Tajik Pamirs [43]. A Bij river sample, collected ∼23 km upstream of the regular weekly sampling point in June 2012 (sample ‘Upper Bij river, in mountains before the dam’, Table 1), showed only slightly lower isotope values (*δ* ^2^H = −137 ‰, *δ* ^18^O = −16.4 ‰) as compared to the three downstream samples from the same period (range from −134.5 to −130.8 ‰ for *δ* ^2^H and −15.6 to −15.2 ‰ for *δ* ^18^O values, Table SM 2), suggesting only a slight isotopic downstream enrichment as a result of evaporation.

The overall range of Ovchuu river samples is small, from −155 to −148 ‰ for *δ* ^2^H values and from −18.6 to −17.1 ‰ for *δ* ^18^O values (linear correlation: *δ* ^2^H = (5.35 ± 0.62)*δ* ^18^O − (56.27 ± 11.14), *R* ^2^ = 0.95, *n* = 5; Figure 4(d), Table 1). Similar to the Bij river, the Ovchuu river samples mainly fall outside and to the left of the 95 % CI of the LMWL, and only the ‘Upper Ovchuu river’ sample, taken 365 m higher upstream falls within the 95 % CI of the LMWL, but has only slightly lower delta and *d*-excess values compared to the four downstream samples.

#### 3.2.2. Other water bodies

The overall *δ* ^2^H and *δ* ^18^O values of analysed water bodies ranged from −168 to −15 ‰ for *δ* ^2^H and from −20.0 to −4.9 for *δ* ^18^O (Table 1), with springs showing lowest enrichment (or highest depletion), and water bodies with standing water and open surfaces (e.g. mountain lakes, small water pools) showing highest enrichment (linked with lowest *d*-excess).

Water bodies with *δ* ^2^H and *δ* ^18^O values fitting the 95 % CI of the LMWL suggest that they are being fed by local precipitation (Figure 4(b)). The enriched *δ* ^2^H and *δ* ^18^O values of individual surface water bodies (e.g. mountain lakes or other standing water pools) to the upper right and outside of the observed 95 % CI of the LMWL (Figure 4(b)) clearly indicate that evaporation had taken place [42,44]. The depleted *δ* ^2^H and *δ* ^18^O values of some surface waters, for example Shiiriin us and individual spring samples (Figure 4(b)) to the left and outside of the observed 95 % CI of the LMWL, suggest that these waters are fed by groundwater sources rather than directly by precipitation.

As the majority of the presented water bodies were only sampled once, it remains unknown how the *δ* ^2^H and *δ* ^18^O values of these water bodies might change over time.

## 4. Conclusions

We present the findings of the first field-based study of *δ* ^2^H and *δ* ^18^O isotope values of precipitation, rivers and other water bodies in the extreme arid environment of the Dzungarian Gobi in Mongolia, and give baseline information for further studies in this area. Isotope values in precipitation showed an extreme range and a high seasonal variability with higher and more variable values in summer and lower values in winter. High variability could not be explained by different origin of air masses alone, but is likely a result of a combination of different processes affecting the isotope values of precipitation in this area. More detailed studies based on long-term field-based data are needed for a better understanding of the importance and the extent of individual processes affecting the *δ* ^2^H and *δ* ^18^O values in precipitation, surface and ground water in this extreme, largely understudied arid Gobi environment.

Our calculated field-based LMWL showed isotopic characteristics of precipitation in an arid region. The observed differences between the field based and modelled (OIPC) *δ* ^2^H and *δ* ^18^O isotope values highlighted the difficulty of modelling the *δ* ^2^H and *δ* ^18^O values for areas with such extreme climatic conditions (i.e. high seasonality, great variability of precipitation in time and space). The lack of long-term GNIP data across large portions of the broader region can probably explain the observed differences between field based and modelled data and thus between the field based and modelled LMWL, which emphasizes the importance of collecting long-term field-based data.

## Supplementary Material

Supplementary_material_sturm_str_HR.docx
