# Supplementary material for: First field-based observations of δ 2H and δ 18O values of event-based precipitation, rivers and other water bodies in the Dzungarian Gobi, SW Mongolia
Source: Isotopes Environ Health Stud. 2016 Oct 12;53(2):157–71. doi: 10.1080/10256016.2016.1231184 (PMC5332016; doi:10.1080/10256016.2016.1231184)
Supplement: Supplementary_material_sturm_str_HR.docx [file gieh_a_1231184_sm8201.docx]

Supplemental Material

Table SM 1: *δ*^2^H, *δ*^18^O and *d*-excess values of precipitation, collected at the Takhin Tal research camp between June 2012 and August 2013, and corresponding OIPC calculated values.

Table SM 2: *δ*^2^H, *δ*^18^O and *d*-excess values of Bij river, collected between June 2012 and September 2013.
